# Quinoxalinones as A Novel Inhibitor Scaffold for EGFR (L858R/T790M/C797S) Tyrosine Kinase: Molecular Docking, Biological Evaluations, and Computational Insights

**DOI:** 10.3390/molecules27248901

**Published:** 2022-12-14

**Authors:** Utid Suriya, Panupong Mahalapbutr, Watchara Wimonsong, Sirilata Yotphan, Kiattawee Choowongkomon, Thanyada Rungrotmongkol

**Affiliations:** 1 Program in Biotechnology, Faculty of Science, Chulalongkorn University, Bangkok 10330, Thailand; 2 Department of Biochemistry, Center for Translational Medicine, Faculty of Medicine, Khon Kaen University, Khan Kaen 40002, Thailand; 3 Department of Chemistry, Center of Excellence for Innovation in Chemistry, Faculty of Science, Mahidol University, Rama VI Road, Bangkok 10400, Thailand; 4 Department of Biochemistry, Kasetsart University, Bangkok 10900, Thailand; 5 Department of Biochemistry, Center of Excellence in Structural and Computational Biology, Chulalongkorn University, Bangkok 10330, Thailand; 6 Program in Bioinformatics and Computational Biology, Graduate School, Chulalongkorn University, Bangkok 10330, Thailand

**Keywords:** EGFR tyrosine kinase, EGFR (L858R/T790M/C797S) TK, lung cancer, non-small cell lung cancer, in silico drug discovery and development

## Abstract

Combating acquired drug resistance of EGFR tyrosine kinase (TK) is a great challenge and an urgent necessity in the management of non-small cell lung cancers. The advanced EGFR (L858R/T790M/C797S) triple mutation has been recently reported, and there have been no specific drugs approved for this strain. Therefore, our research aimed to search for effective agents that could impede the function of EGFR (L858R/T790M/C797S) TK by the integration of in silico and in vitro approaches. Our in-house quinoxalinone-containing compounds were screened through molecular docking and their biological activity was then verified by enzyme- and cell-based assay. We found that the four quinoxalinone-containing compounds including CPD4, CPD15, CPD16, and CPD21 were promising to be novel EGFR (L858R/T790M/C797S) TK inhibitors. The IC_50_ values measured by the enzyme-based assay were 3.04 ± 1.24 nM; 6.50 ± 3.02 nM,10.50 ± 1.10 nM; and 3.81 ± 1.80 nM, respectively, which are at a similar level to a reference drug; osimertinib (8.93 ± 3.01 nM). Besides that, they displayed cytotoxic effects on a lung cancer cell line (H1975) with IC_50_ values in the range of 3.47 to 79.43 μM. In this proposed study, we found that all screened compounds could interact with M793 at the hinge regions and two mutated residues including M790 and S797; which may be the main reason supporting the inhibitory activity in vitro. The structural dynamics revealed that the screened compounds have sufficient non-native contacts with surrounding amino acids and could be well-buried in the binding site’s cleft. In addition, all predicted physicochemical parameters were favorable to be drug-like based on Lipinski’s rule of five, and no extreme violation of toxicity features was found. Altogether, this study proposes a novel EGFR (L858R/T790M/C797S) TK inhibitor scaffold and provides a detailed understanding of compounds’ recognition and susceptibility at the molecular level.

## 1. Introduction

Cancers pose a public health burden and challenge to all nations and remain a major cause of mortality and morbidity. Lung cancer is the most common type of cancer worldwide. It has been reported that more than 2.2 million were new cases; among these non-small cell lung cancer (NSCLC) accounts for 84% of all newly diagnosed cases (https://www.cancer.org/, accessed on 30 October 2022). One of the major advancements in the treatment of NSCLC came with the development of drugs inhibiting the biomarkers which could be produced by tumors or our body in response to the malignancy. Specifically targeting the up-regulated kinases such as the epidermal growth factor receptor tyrosine kinase (EGFR TK) and interrupting the signaling intermediates such as mitogen-activated protein kinase (MAPK) and Janus kinase (JAK)/STAT have been a promising strategy to reverse the rate of cancer-related mortality.

**Figure 1:**
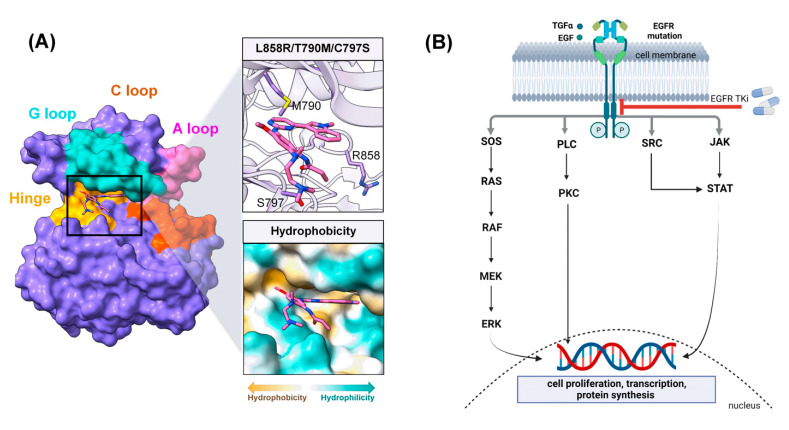
(**A**) 3D structure of EGFR (L858R/T790M/C797S) TK in complex with osimertinib (PDB ID: 6LUD) in which the mutated amino acids were shown in a close-up view as well as a hydrophobic character of its binding cleft. (**B**) EGFR signaling pathways including Sos/Ras/Raf/MEK/ERK, PLC/PKC, and JAK/STAT signaling cascade that control cell differentiation, proliferation, and survival responses [4,33]. (adapted from Refs. [4,33] and created with https://BioRender.com, accessed on 30 October 2022).

EGFR TK, one of the members of the ErbB family, is a transmembrane glycoprotein that plays a pivotal role in the regulation of cellular proliferation, homeostasis, growth, and survival responses [1]. Structurally, the EGFR TK domain consists of four main elements [2] including hinge region (residues 788–797), P loop (residues 712–731), C helix (residues 752–767), and activation loop (855–877) as illustrated in Figure 1. The signal transduction begins when EGF binds to the ligand binding site and triggers homodimerization or heterodimerization with other ERBB members (e.g., HER2). Then, the receptor phosphorylates and activates downstream effectors such as RAS–RAF–MEK–ERK–MAPK and PI3K–AKT–mTOR signaling cascade [3,4]. Biological proven shreds of evidence have revealed the correlation between the overexpression of EGFR and human malignancies leading to the enhancement of tumor cell proliferation, growth, invasion, metastasis, angiogenesis, and even apoptosis impairment in several types of cancers such as breast cancer, head and neck cancer, lung cancer, etc. [5,6,7,8]. To this end, several pharmaceutical agents have been developed for therapeutic purposes and now become a standard treatment for patients with EGFR-positive non-small-cell lung cancer (NSCLC). Undoubtedly, similar to other proteins, mutations have been reported during the long-term drug administration [9], which include a single-point mutation in exon 21 (L858R) and exon-19 deletion, L858R/T790M double mutation, and L858R/T790M/C797S triple mutation. Accordingly, currently available drugs have been designed to fight against acquired drug resistance, which can be classified into three generations: (i) the first generation: erlotinib [10] and gefitinib [11,12,13], which belong to the quinazoline-based scaffold (ii) the second generation: afatinib and dacomitinib were developed to overcome acquired drug resistance caused by T790M mutation [14,15,16] (iii) the third generation: WZ-4002 [17], CO-1686 (reciletinib) [18], and osimertinib [19] has been developed to potently and specifically inhibit L858R/T790M double mutated EGFR. Nevertheless, there has been reported that approximately 40% of NSCLC patients have inevitably developed triple mutation, promoting a loss of inhibitory activity of previously approved agents [20]. Only osimertinib, the FDA- and EMA-approved third-generation inhibitor, remains the first-line therapy and standard treatment for NSCLC patients carrying EGFR mutations [19,21,22]. A rising incidence of drug resistance caused by the advanced L858R/T790M/C797S triple mutation in NSCLC escalates the urge for the development of the fourth generation of EGFR inhibitors, which aim to effectively deliver a significant clinical response in patients with a triple mutation.

There are several potent compounds capable of inhibiting EGFR TK such as trisubstitued imidazoles [23], trisubstituted pyridinylimidazoles [24], 9-heterocyclyl substituted 9H-purine derivatives [25], quinoline derivatives [26], methylpyrimidopyridone scaffold [27], pyrimidine derivatives [28], N^2^-(4-(4-methylpiperazin-1-yl) phenyl)-N^8^-phenyl-9H-purine-2, 8-diamine [29], sulfonyl fluoride derivatives [30], anilino-1,4-naphthoquinones [31], etc. Quinoxalinones have broad-ranging pharmacological activities including anticancer properties [32]; however, there has been no report of the specific binding of quinoxalinones toward EGFR (L858R/T790M/C797S) triple mutant. Herein, we performed in silico screening of in-house quinoxalinone derivatives which were successfully synthesized by phenyliodine (III) diacetate (PIDA)-induced oxidative C-N bond coupling. Compounds exhibiting a comparable level of binding affinity to osimertinib were then selected to be tested experimentally. The potential compounds were predicted for their drug-likeness properties and toxicity features. Then, the MD simulations were employed to gain insights into binding recognition and susceptibility, guiding the mechanism of inhibitory action at the atomic level. Altogether, our proposed quinoxalinone derivatives could become viable candidates to be further developed as the effective fourth generation of EGFR inhibitors.

## 2. Results and Discussion

### 2.1. Docking-Based Virtual Screening

**Figure 2:**
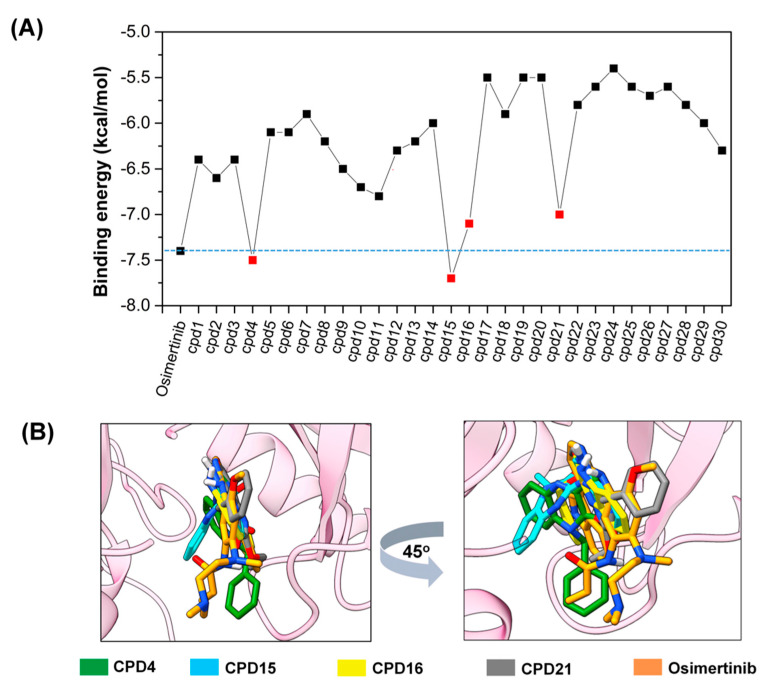
Docking-based virtual screening of 30 quinoxalinone-containing compounds in comparison to the known drug, osimertinib. (**A**) The four compounds possessing a lower or equal to −7.0 kcal/mol were then selected to perform experiments as highlighted in red. (**B**) Superimposition of the docked pose of all screened compounds and osimertinib.

**Figure 3:**
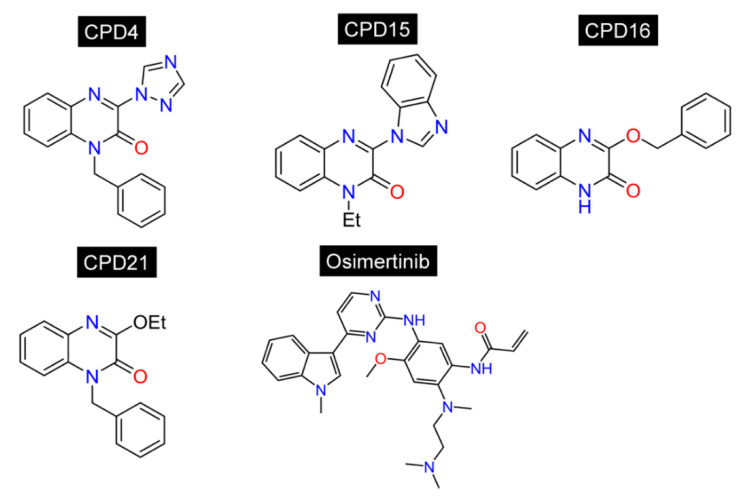
The chemical structures of selected compounds including CPD4, CPD15, CPD16, and CPD21 as well as a reference drug (osimertinib).

To rapidly identify the potential compounds from 30 in-house quinoxalinones capable of binding to the TK domain of EGFR (L858R/T790M/C797S), molecular docking was performed by employing the Autodock Vina XB software package [34,35]. As shown in Figure 2, the predicted binding energy of quinoxalinone-containing compounds was in a range −7.8 to −5.4 kcal/mol whilst the osimertinib drug was predicted to be −7.4 kcal/mol. Selection of potential compounds was based on the predicted binding energy, which is theoretically proportional to the dissociation constant (k_d_) and is widely used in several computer-aided drug discovery campaigns. For this purpose, compounds showing binding energy lower than −7.0 kcal/mol were selected which resulted in four quinoxalinone-containing compounds including CPD4, CPD15, CPD16, and CPD21. Note that their chemical structures are shown in Figure 3.

### 2.2. In Vitro Assay of EGFR (L858R/T790M/C797S) Inhibition

**Figure 4:**
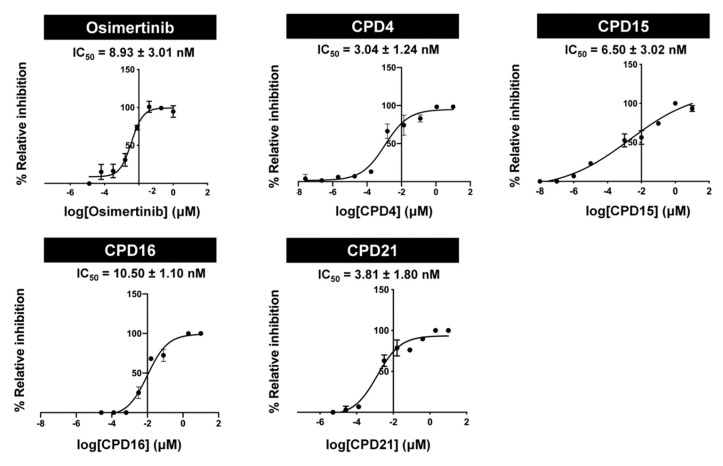
Dose–response curves of kinase inhibitory activity against EGFR (L858R/T790M/C797S) TK. Data were represented as mean ± SEM.

The screened compounds from molecular docking were assayed for kinase inhibitory activity, which was reported as half-maximal inhibitory concentration (IC_50_) in comparison to the known drug, osimertinib. As shown in Figure 4, the kinase inhibitory activity against EGFR (L858R/T790M/C797S) was in a nanomolar scale in which the IC_50_ values of CPD4, CPD15, CPD16, and CPD21 were 3.04 ± 1.24 nM, 6.50 ± 3.02 nM, 10.50 ± 1.10 nM, and 3.81 ± 1.80 nM, respectively, while osimertinib showed an IC_50_ value of 8.93 ± 3.01 nM. Statistical analysis (Tukey’s test) showed no significant difference in IC_50_ value when compared to osimertinib at 95% confidence; thus, CPD4, CPD15, CPD16, and CPD21 could be very potent inhibitors as similar as the approved drug, osimertinib. The inhibitory activity of these screened compounds was found to be similar to several previously reported compounds such as trisubstitued imidazoles (20–300 nM) [23], 9-heterocyclyl substituted 9H-purine derivatives (18 nM) [25], quinoline derivatives (115–139 nM) [26], methylpyrimidopyridone scaffold (27.5 nM) [27], sulfonyl fluoride derivative (110 nM) [30], anilino-1,4-naphthoquinones (3.96–18.64 nM) [31], etc.

### 2.3. Drug-Likeness

The potent quinoxalinone-containing compounds against EGFR (L858R/T790M/C797S) were predicted the drug-likeness character based on Lipinski’s rule of five by inspecting their physicochemical properties (molecular weight (MW), the numbers of hydrogen bond donors (HBD) and acceptors (HBA), rotatable bond (RB), polar surface area (PSA) and Log *P*). According to Table 1, the predictive results revealed that all compounds could confer the drug-likeness property and obey the acceptable value within the criteria of the rules as follows: (i) molecular weight ≤ 500 Da, (ii) hydrogen bond donors ≤5, and hydrogen acceptors ≤10, (iii) rotatable bond ≤10, (iv) polar surface area ≤140 Å^2^ and (v) lipophilicity (expressed as Log *P*) ≤5. Therefore, we believed that these four compounds were likely to be developed as promising novel mutated EGFR inhibitors.

### 2.4. SASA, Number of H-Bonds, and Contact Atoms

**Figure 5:**
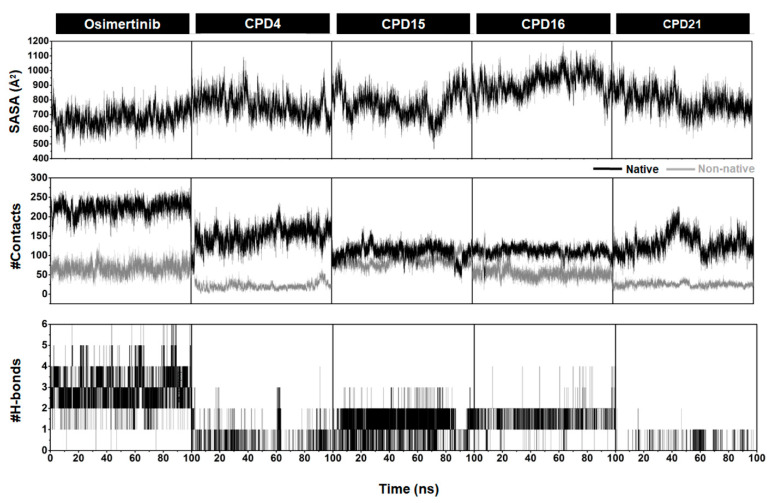
Plots of SASA and contact atoms counted within the 5.0 Å from the ligand as well as number of H-bonds by setting two criteria as follows: (1) the distance between the hydrogen bond donor (HD) and hydrogen acceptor (HA) of ≤3.5 Å (2) the angle ≥120°.

To investigate the buried capacity of each compound within the cleft of the binding site, the solvent-accessible surface area (SASA) was computed by inspecting the amounts of water within the 5.0 Å from the ligand. As shown in Figure 5, the averaged SASA in the last 20 ns (considered reaching an equilibrated state, Figure S4) of each complex was in the order of CPD4 (674 Å^2^) < CPD15 (730 Å^2^) < osimertinib (736Å^2^) < CPD21 (768 Å^2^) < CPD16 (866 Å^2^). The smaller area of occupied solvents could imply the better-fitting ligand conformation within the binding pocket and the binding events might not be interfered with by the solvents. Therefore, CPD4 was the most well-buried to binding pocket; however, the intermolecular interactions of CPD16 and surrounding amino acids might be probably reduced. Apart from that, the numbers of H-bonds were analyzed during 80–100 ns. It was found that quinoxalines could form fewer H-bonds when compared to osimertinib (1–2 bonds), suggesting that this type of interaction was not dominantly responsible for the quinoxalinone compounds’ recognition since they comprise gradually lower numbers of hydrogen bond donors and acceptors when compared to osimertinib (total numbers of HBD and HBA are 3–4 while there are 7 for osimertinib, Table 1).

We also identified the number of contact atoms within the 5.0 Å from the ligand. As shown in Figure 5, the native contacts of quinoxalinones were less than osimertinib. Nevertheless, the non-native contacts demonstrated a similar range falling onto 50–80 atoms (last 20 ns), indicating that the screened compounds could interact with the surrounding amino acids when the dynamics was taken into account. In the case of osimertinib, due to its structure being larger than quinoxalinones (1.5 to 2-fold larger molecular weight, Table 1), it could plausibly expose to a larger number of proximate amino acids when compared to quinoxalinones.

### 2.5. Hot-Spot Residues

**Figure 6:**
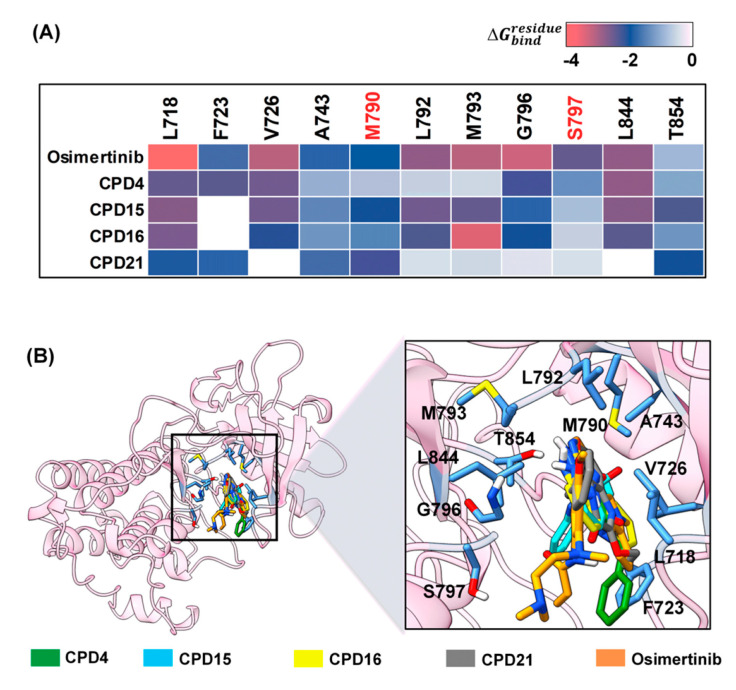
Binding free energy decomposed to each residue (only some key influential amino acids were shown). (**A**) The level of residue contribution was shaded in a red-blue-white color scale, which was ranging from higher to lower contribution, respectively. Note that the mutated amino acids were shown in red word. (**B**) A close-up view of key influential amino acids contributing to ligand binding in complex with screened compounds and osimertinib.

To investigate the key binding residues within the focused site of EGFR tyrosine kinase, the per-residue decomposition energy (${∆G}_{\mathrm{residue}}^{\mathrm{bind}}$) was elucidated via the MM/PBSA approach. The positive and negative ${∆G}_{\mathrm{residue}}^{\mathrm{bind}}$ values represent the ligand destabilization and stabilization, respectively. We noted that only residues exhibiting ${∆G}_{\mathrm{residue}}^{\mathrm{bind}}$ value lower than −0.10 kcal/mol were elucidated and compared with other residues. As seen in Figure 6, quinoxalinones showed somewhat a similar pattern of binding amino acids compared to osimertinib. The common amino acids participating in the ligand binding include L718, F723 (except CPD15), V726 (except CPD21), A743, M790, L792, G796, S797, L844, and T854 (except CPD21), which agreed well with the previous report [36]. The amino acids largely contributing to ligand binding consist of both G loop and hinge region. The L718 and V726 (except CPD21) were found to noticeably influence to the binding in almost all screened compounds and osimertinib, which agreed well with the previous findings [36]. In addition, the M793 lining at the hinge region was detected in all screened compounds’ binding, which has been highlighted as a satisfied condition of successful EGFR TK inhibitors by controlling the entry of inhibitors to the ATP-binding pocket [36,37]. Interestingly, all screened compounds could interact with two mutated residues including M790 and S797, which are lining in the ligand binding site. It is worth noting that CPD16 showed higher binding energy to M793 than osimertinib, revealing a satisfied condition of being successful EGFR TK inhibitors. Altogether, this finding could provide an explanation in terms of structural basis that support the great inhibitory activity in vitro of all screened compounds.

**Figure 7:**
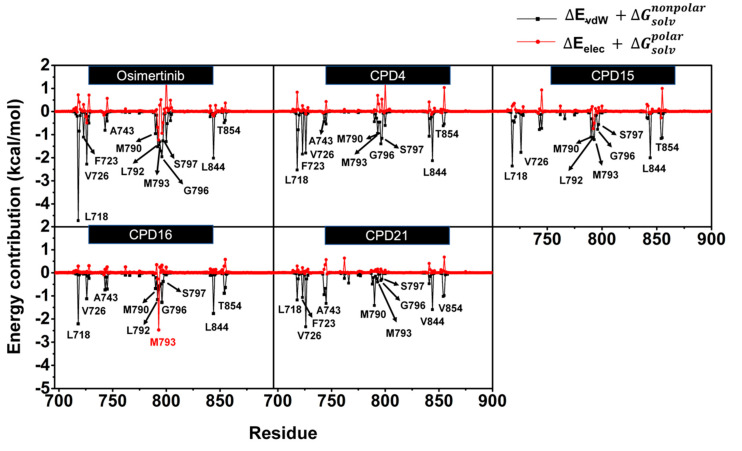
Calculated per-residue vdW (∆E_vdW_ + $∆G_{sol}^{nonpolar}$) and electrostatic (∆E_elec_ + $∆G_{sol}^{polar}$) decomposition energy to each amino acid largely contributed to ligand binding.

In addition, the energy contributions to each amino acid were observed. All screened compounds shared somewhat a similar pattern to osimertinib in which the most amino acids were recognized through van der Waals interaction energies ($∆E_{\mathrm{vdW}}+∆G_{\mathrm{solv}}^{\mathrm{nonpolar}}$) while the electrostatic term ($∆E_{\mathrm{elec}}+∆G_{\mathrm{solv}}^{\mathrm{polar}}$) was not preferably dominant toward the binding (Figure 7). This finding agreed well with the previous study that suggested a deep hydrophobic pocket within the ATP-binding site of EGFR TK that is controlled by the residue at 790 position (known as a gatekeeper) [2]. However, CPD16 showed a higher electrostatic contribution to M793 than other quinoxalinones and even osimertinib, suggesting this compound could be well recognized during the binding event. Interestingly, the residue T854 demonstrated a higher contribution of van der Waals interaction energies to all screened compounds when compared to osimertinib, implying the main distinct character of inhibitory actions of quinoxalinones.

### 2.6. Toxicity Prediction

Toxicity is one of the crucial factors that has been taken into consideration prior to continuing the next phase of drug discovery campaigns. Herein, we employed the available databases to predict the main crucial features necessary for inspecting compounds’ toxicity. As listed in Table 2, all screened compounds showed no predicted mutagenicity, tumorigenicity, irritant, and reproductive effects (except CPD4; low level) as well as the predicted LD_50_ values were in the range of slightly toxic (5000 mg/kg > LD50 > 500 mg/kg). For osimertinib, it was predicted to be highly prone to mutagenicity and was moderately toxic (500 mg/kg > LD50 > 50 mg/kg). Hence, all screened quinoxalinones were predicted to be safe and might reduce the rate of failure caused by toxicity.

### 2.7. Effect of CPD Derivatives on the Cell Viability

The cytotoxic effect of CPD derivatives on H1975 lung cancer cells expressing L858R/T790M EGFR as well as Vero normal kidney cells was evaluated using MTT assay. Osimertinib, a third-generation EGFR TKi used to treat adenocarcinoma patients carrying EGFR mutation [22], was used as the positive control. As shown in Table 3, all CPD derivatives as well as osimertinib inhibited the growth of H1975 cells in a concentration-dependent manner. Of all CPD derivatives, CPD4 exhibited the most potent cytotoxicity toward H1975 cells (IC_50_ = 3.47 ± 2.20 μM), followed by osimertinib (IC_50_ = 18.33 ± 2.02 μM), CPD15 (IC_50_ = 31.25 ± 3.40 μM), CPD21 (IC_50_ = 44.67± 2.34 μM), and CPD16 (IC_50_ = 79.43± 4.35 μM). Interestingly, all CPD derivatives, especially CPD15, CPD16, and CPD21 exhibited low cytotoxic effects on Vero normal kidney cells (IC_50_ > 100 μM). These results suggested that CPD derivatives are promising anticancer agents with high safety profiles. We noted that the cell viability of H1975 and Vero was shown in Figure S5 and Figure S6, respectively.

## 3. Materials and Methods

### 3.1. Docking-Based Virtual Screening

In this study, protein-ligand docking was performed using the crystal structure of EGFR (L858R/T790M/C797S) in a complex with the known drug, osimertinib (PDB ID: 6LUD [38]). The missing residues (No. 748–751 and 991–999) were homologically built using the SWISS-MODEL server [39]. The newly constructed protein structure was then validated by plotting a Ramachandran diagram using PROCHECK [40]. In addition to the protein structure, the quinoxalinones synthesized by Wimonsong et.al., [41] were manually constructed into 3D structures by using the Gaussian 09 program [42].

For docking validation, the crystalized ligand was defined as a center in the binding site, and redocked into the same binding site, which was in X, Y, and Z coordinates as of −48.818, −2.761, and −18.500, respectively, while the box size was set to 40 Å for every dimension. The crystallized and redocked poses were then aligned to observe the verification of the docking protocol used as shown in Figure S3. Accordingly, the validated docking protocol was employed for all ligands during virtual screening. The docking-based screening was executed by the Autodock Vina XB software package (Sirimulla Researcg Group@University of Texas at EI Paso, EI Paso, TX, USA).

### 3.2. EGFR Tyrosine Kinase Inhibition

The inhibitory activity of each candidate compound towards EGFR triple mutations was performed using the luminescent ADP-Glo™ kinase assay (Promega Corporation, Madison, WI, USA) as previously described [43,44]. According to the reaction recipe, 8 μL of buffer (40 mM Tris-HCl pH 7.5, 20 mM MgCl_2_ and 0.1 mg mL^−1^ bovine serum albumin) was added to a 384-well plate, followed by 5 μL of enzyme (1.25 ng μL^−1^). Then, 2 μL of inhibitors and 10 μL of a mixture containing 25 μM ATP and 2.5 μM poly(glu-tyr) were added and the reaction was incubated for 1 h at room temperature, followed by adding 5 μL of the ADP-Glo reagent and the reaction was further incubated for 40 min. Subsequently, 10 μL of kinase detection reagent was added and incubated at room temperature for 30 min to convert ADP into ATP. The generated luminescence by ATP in a luciferase reaction was then measured using a microplate reader (Infinite M200 microplate reader, Tecan, Männedorf, Switzerland), which corresponds to kinase activity. All assays were performed in triplicate and the obtained data were represented as the relative inhibition (%) of inhibitors compared to the control with no inhibitor as shown in Equation (1) [45];

(1) $$\%\;\mathrm{relative}\;\mathrm{inhibition}=\frac{\left[\left(\mathrm{positive}-\mathrm{negative}\right)-\left(\mathrm{sample}-\mathrm{negative}\right)\right]}{\left(\mathrm{positive}-\mathrm{negative}\right)}\times 100$$

### 3.3. Molecular Dynamic (MD) Simulations

MD simulations were applied to gain insights into the key binding residues guiding the mechanism of inhibitory action in the dynamical and physiological system. The initial structure of each EGFR (L858R/T790M/C797S) TK and focused ligand was retrieved from the best docking pose (lowest E_binding_). Then, the constructed complex was subjected to run under the periodic boundary condition with the isothermal-isobaric (NPT) scheme (310 K, 1 atm), according to the previous studies [35,36,46,47]. The AMBER ff14SB force field and the generalized AMBER force field version 2 (GAFF2) were applied to treat bonded and non-bonded interaction parameters of the simulated complex [48] whilst the water model was used to solvate the system [49]. To neutralize the overall charge of the system, either sodium or chloride ion was then randomly introduced according to the calculated numbers of charges. Minimization of the hydrogen atoms and water molecules was carried out using 500 steps of steepest descent (SD) followed by 1500 steps of conjugated gradient (CG) methods while the rest of the molecules were fixed. Next, the minimization of the protein-ligand complex (constrained solvents) and the whole complex system was minimized again by using the same procedure, according to the previous publications [30,50,51].

The particle mesh Ewald summation approach [52] was used to handle electrostatic interactions, while all hydrogen atoms were constrained by the SHAKE algorithm [53]. The temperature was heated from 10 to 310 K by using a Langevin thermostat [54] with a collision frequency of 2 ps^−1^ whilst the pressure was created by the Berendsen barostat [55]. The MD production lasted to 100 ns by the 2 fs increment of a time step. The MD outputs were elucidated through the cpptraj module, while the per-residue decomposition energy (${∆G}_{\mathrm{binding}}^{\mathrm{residue}}$) was computed by MM/PBSA.py implemented in AMBER16.

### 3.4. Pharmacokinetic Properties Prediction

The focused pharmacokinetic properties including physicochemical characteristics and drug-likeness were predicted by using the web-based program SwissADME (SIB Swiss Institute of Bioinformatics, Écublens, Switzerland, www.swissadme.ch/, accessed on 30 October 2022) [56]. The screened compounds’ features were calculated compared to the known drug, osimertinib.

### 3.5. Synthesis and Characterization of CPD21

**Scheme 1:**
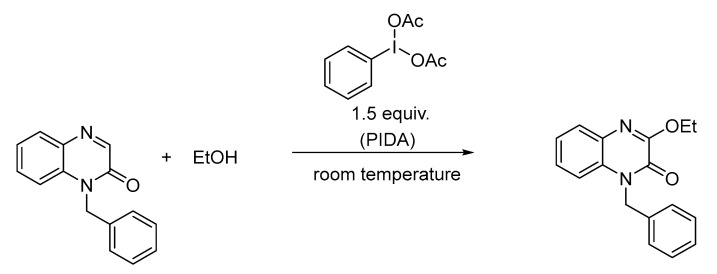
Synthesis of CPD21.

Since the CPD21 has not been published yet, the synthesized procedure and characterization were described in this study. According to the general procedure [41], 1-Benzyl-3-ethoxyquinoxalin-2(1*H*)-one (CPD21) was synthesized from benzylquinoxalin-2(1*H*)-one (118.1 mg, 0.50 mmol) in ethanol (2 mL) as shown in Scheme 1. Column chromatography (silica gel; EtOAc/Hexane, 1:1) gave the title compound 1-benzyl-3-ethoxyquinoxalin-2(1*H*)-one as a white solid (251 mg, 90% yield). ^1^H NMR (CDCl_3_, 400 MHz): δ 7.61 (dd, *J* = 7.4, 1.8 Hz, 1H), 7.31–7.17 (m, 8H), 5.51 (s, 2H), 4.57 (q, *J* = 7.1 Hz, 2H), 1.52 (t, *J* = 7.1 Hz, 3H) ppm, ^13^C NMR (CDCl_3_, 100 MHz): δ 154.1, 151.6, 135.3, 131.6, 130.9, 129.0, 127.8, 127.7, 127.1, 127.0, 124.1, 114.6, 63.7, 46.4, 14.3 ppm, and HRMS (ESI): m/z [M + H]^+^ calcd for C_17_H_16_N_2_O_2_: 281.1285; found: 281.1283 (Figures S1 and S2). For other screened compounds, their synthesis scheme was described in this paper [37] as listed in Table S1.

### 3.6. Toxicity Prediction

In silico profiling of toxicity featured (mutagenicity, tumorigenicity, irritant, and reproductive effect) was performed by using the DataWarrior software package (version 5.5.0, Thomas Sander, https://openmolecules.org, accessed on 30 October 2022, [57]). The prediction of lethal dose at 50% (LD_50_) and the toxicity class were performed by a web-based Protox-II server [58] (Available online, https://tox-new.charite.de/protox_II/, accessed on 30 October 2022).

### 3.7. Cell Lines, Culture, and Cytotoxicity Assay

Human NSCLC cell lines, H1975 (ATCC CRL-5908) and monkey’s normal kidney epithelial cell line (Vero, ATCC CCL-81) were purchased from American Type Culture Collection (ATCC, Manassas, VA, USA). H1975 cells were grown in RPMI-1640 medium supplemented with 10% FBS, 100 U/mL penicillin, and 100 µg/mL streptomycin. Vero cells were grown in Dulbecco’s modified Eagle’s minimal essential medium (DMEM; Gibco, Grand Island, NY, USA) containing 10% fetal bovine serum (FBS; Gibco), 100 U/mL penicillin, and 100 µg/mL streptomycin (Gibco). Both cells were maintained to grow at 37 °C in a humidified 5% CO_2_ atmosphere.

The studied cell lines were seeded into 96-well plates (cell density of 6000 cells/well) and were incubated overnight. Then, cells were treated with screened compounds and the known drug with various concentrations (125, 62.5, 31.25, 15.63, 7.81, 3.91, 1.95, and 0.98 μM) for 72 h. After incubation, the effect of compounds on cell viability was measured using the MTT assay as follows: 10 μL of MTT solution (5 mg/mL) was added and then incubated for 3 h. The medium was removed, followed by adding 50 μL of DMSO to dissolve the crystal formazan product. The purple solution of crystal formazan was then measured at 570 nm using a microplate spectrophotometer (Infinite M200 microplate reader, Tecan, Männedorf, Switzerland). The complete medium with 1.0% DMSO (no inhibitor) was used as a control in accordance with the reaction containing 1.0% DMSO of all wells.

### 3.8. Statistical Analysis

The data was represented as mean ± standard error of the mean (SEM). Differences between groups were compared using one-way ANOVA (Tukey’s test) for multiple comparisons. The differences in means were determined at the confidence level *p* ≤ 0.05.

## 4. Conclusions

Finding pre-clinical drug candidates against acquired drug resistance of EGFR tyrosine kinase is a great challenge and necessary. Herein, synthesized quinoxalinone-containing compounds were explored through in silico and in vitro methods. From screening to biological assay, CPD4, CPD15, CPD16, and CPD21 showed great inhibitory activity in enzymatic assay with IC_50_ values down to a nanomolar scale as being a comparable level when compared to the known drug, osimertinib. They could also inhibit the NSCLC cell growth in a concentration-dependent manner with safety profiles. Based on computational modeling, we proposed some common amino-acid residues which are responsible for the binding of all screened compounds and the drug include L718 and V726 at the G loop as well as M790, M793, and S797 at the hinge region. Moreover, all screened compounds could be intermolecularly recognized by two previously mutated amino acids including M790 and S797, which is the main reason to advocate the inhibitory capability against EGFR (L858R/T790M/C797S) TK. In addition, all predicted physicochemical parameters based on Lipinski’s rule of five had met the criteria of drug-likeness and were predicted to show no highly toxic features. Altogether, we proposed that CPD4, CPD15, CPD16, and CPD121 could become viable candidates for development as the fourth generation of EGFR (L858R/T790M/C797S) TK. Animal testing is encouraged to investigate further.

## Figures and Tables

**Table 1:** Predicted Lipinski’s rule of five for the quinoxalinone-based compounds and osimertinib. (HBD, hydrogen bond donor; HBA, hydrogen bond accepter; PSA, polar surface area).

Lipinski’s Rule of Five							
Compounds	M.W. (Da)	HBD	HBA	Rotatable Bonds	PSA (Å^2^)	logP	Drug-Likeness
CPD4	303.32	0	4	3	65.60	2.45	Yes
CPD15	290.32	0	3	2	52.71	2.83	Yes
CPD16	252.27	1	3	3	54.98	2.32	Yes
CPD21	280.32	0	3	4	44.12	3.00	Yes
Osimertinib	499.61	2	5	10	90.78	3.36	Yes

**Table 2:** Prediction of the main toxicity features of all screened quinoxalinones in comparison to osimertinib.

Features	Compounds				
	Osimertinib	CPD4	CPD15	CPD16	CPD21
LD_50_ (mg/kg)	100	1000	1000	800	1000
Toxicity class	3	4	4	4	4
Mutagenicity	High	None	None	None	None
Tumorigenicity	Low	None	None	None	None
Irritant	Low	None	None	None	None
Reproductive effect	Low	Low	None	None	None

**Table 3:** Cytotoxic effect of CPD derivatives and osimertinib on the H1975 and Vero cell viability represented as IC_50_ values.

Compounds	IC_50_ (μM)	
	H1975	Vero
CPD4	3.47 ± 2.20	>20
CPD15	31.25 ± 3.40	>50
CPD16	79.43 ± 4.35	>100
CPD21	44.67 ± 2.34	>100
Osimertinib	18.33 ± 2.02	>100

## Data Availability

Not applicable.

## Supplementary Materials

The following are available online at https://www.mdpi.com/article/10.3390/molecules27248901/s1, Table S1: Chemical structures of all synthesized quinoxalinone-containing compounds and their original code in the previously published paper, Figure S1: 1H spectrum of CPD21 (CDCl3, 400 MHz), Figure S2: 13C NMR spectra of CPD21 (CDCl3, 100 MHz), Figure S3: Alignment of the re-docked pose (green) and available crystallized osimertinib (grey) showing the validated docking protocol used to perform docking-based virtual screening, Figure S4: Calculated backbone root-mean-square displacement (RMSD) within 5 Å of all screened compounds and osimertinib, Figure S5: H1975 cell viability against treated screened compounds and osimertinib at different doses. Data were represented as mean ± SEM with three replicates, Figure S6: Vero cell viability against treated screened compounds and osimertinib at different doses. Data were represented as mean ± SEM with three replicates.

## Sample Availability

Samples of the compounds are available from the authors but only with the permission of the Department of Chemistry, Center of Excellence for Innovation in Chemistry, Faculty of Science, Mahidol University.
