# Supplementary material for: Quinoxalinones as A Novel Inhibitor Scaffold for EGFR (L858R/T790M/C797S) Tyrosine Kinase: Molecular Docking, Biological Evaluations, and Computational Insights
Source: Molecules. 2022 Dec 14;27(24):8901. doi: 10.3390/molecules27248901 (PMC9788584; doi:10.3390/molecules27248901)
Supplement: Supplementary file 1 [file molecules-27-08901-s001.zip › molecules-2047655-supplementary.pdf]

Supplementary Materials: Quinoxalinones as A Novel Inhibitor Scaffold for EGFR (L858R/T790M/C797S) Tyrosine Kinase: Molecular Docking, Biological Evaluations, and Computational Insights

Table S1: Chemical structures of all synthesized quinoxalinone-containing compounds and their original code in the previously published paper.

Name	Structure	Original code	Name	Structure	Original code
cpd1	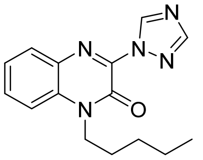 Molecular Weight: 283.3350	3c	cpd11	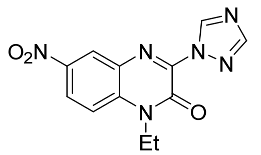 Molecular Weight: 286.2510	3j
cpd2	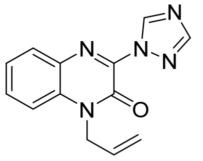 Molecular Weight: 253.2650	3e	cpd12	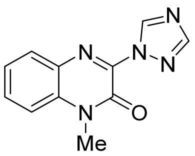 Molecular Weight: 227.2270	3b
cpd3	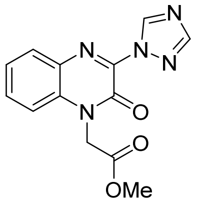 Molecular Weight: 285.2630	3f	cpd13	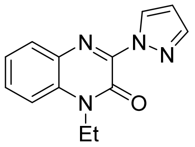 Molecular Weight: 240.2660	3r
cpd4	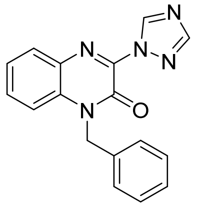 Molecular Weight: 303.3250	3d	cpd14	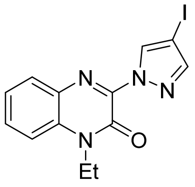 Molecular Weight: 366.1625	3t
cpd5	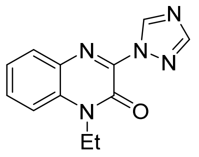 Molecular Weight: 241.2540	3a	cpd15	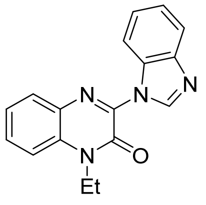 Molecular Weight: 290.3260	3o

cpd8		3m	cpd16		5c
cpd9		3n	cpd21		N/A
cpd10		3l			

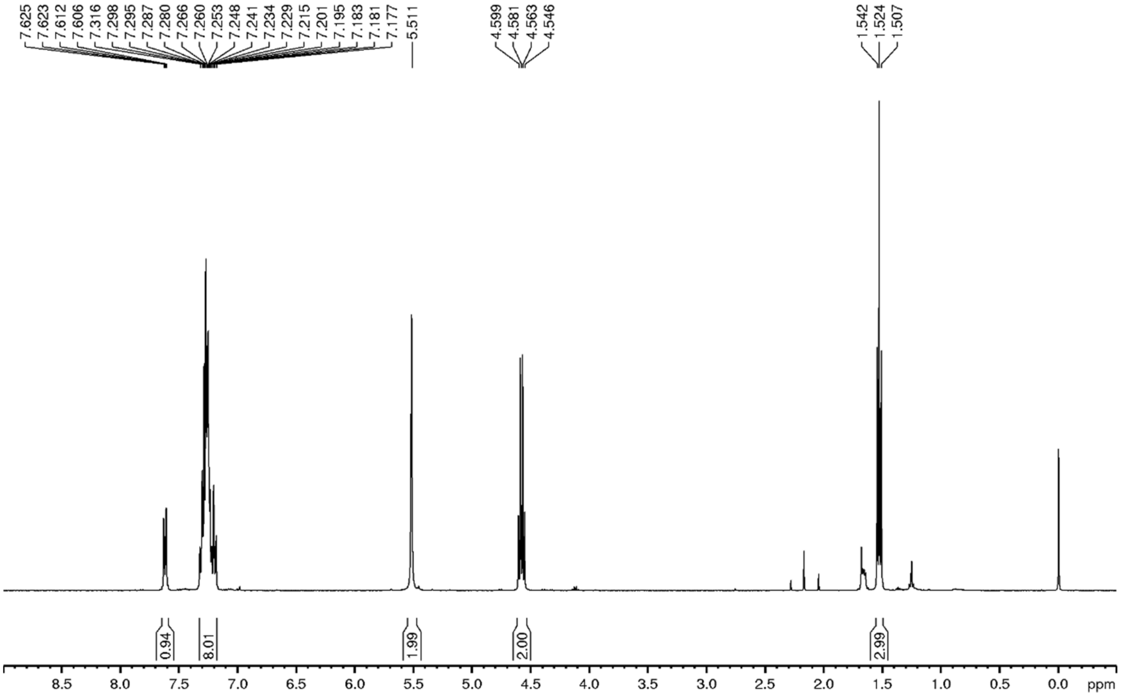

Figure S1: ^{1}H spectrum of CPD21 ($CDCl_3$, 400 MHz).

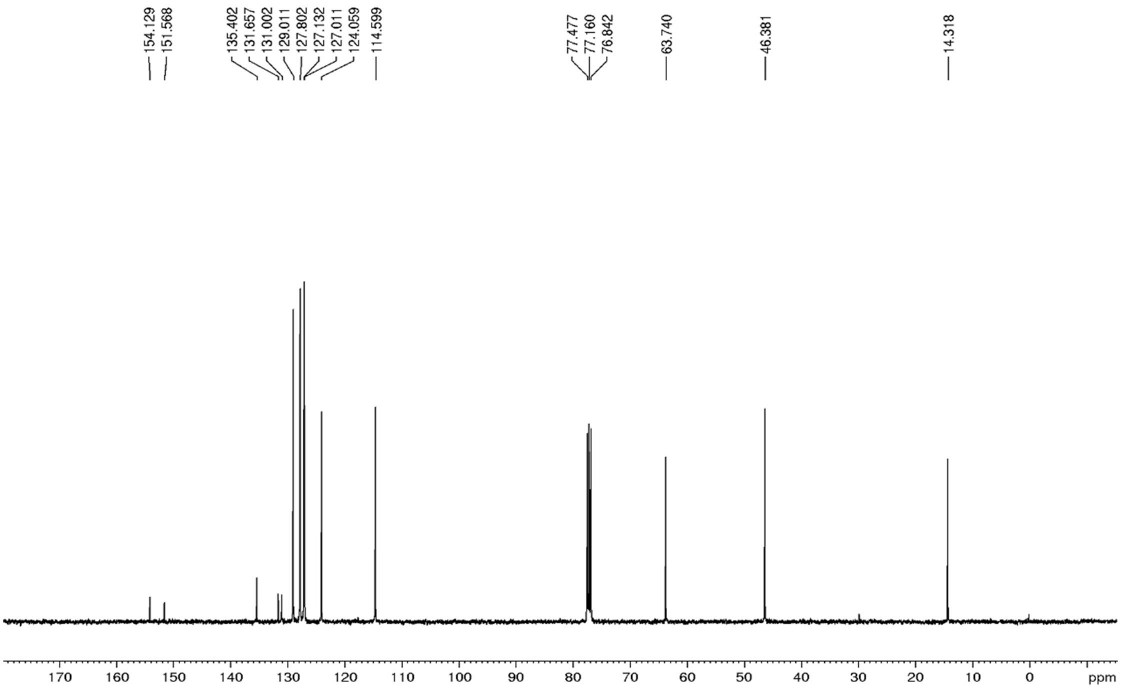

Figure S2: ^{13}C NMR spectra of CPD21 ($CDCl_3$, 100 MHz).

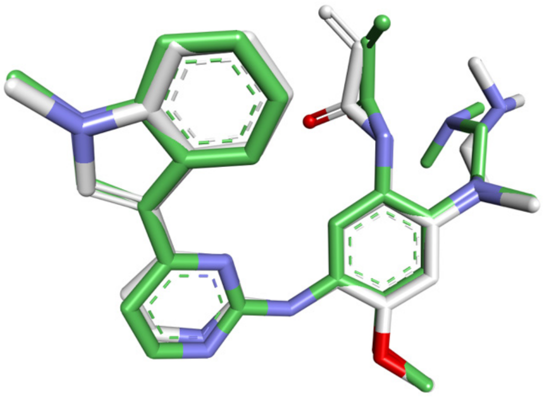

Figure S3: Alignment of the re-docked pose (green) and available crystallized osimertinib (grey) showing the validated docking protocol used to perform docking-based virtual screening. We noted that the N- and O-atom were shaded in purple and red color, respectively.

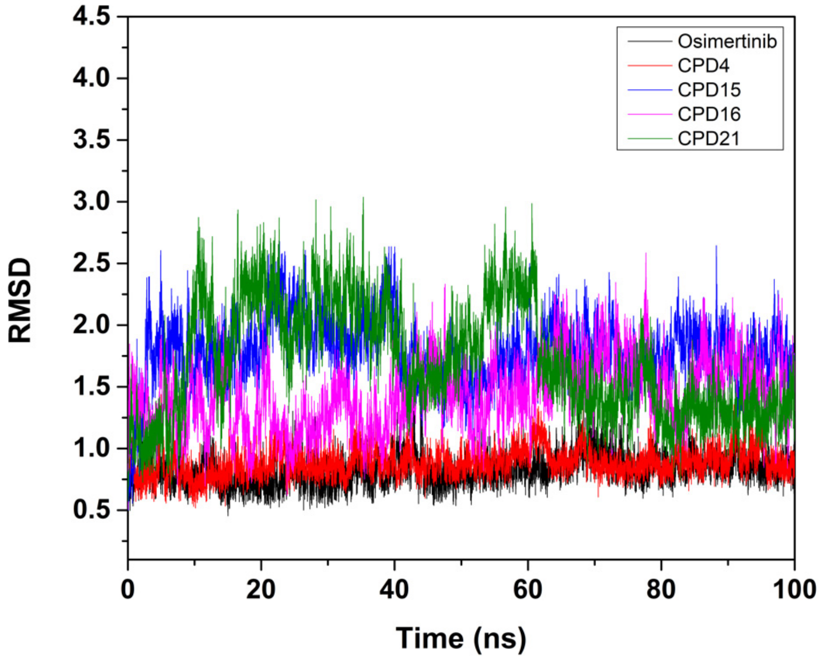

Figure S4: Calculated backbone root-mean-square displacement (RMSD) within 5 Å of all screened compounds and osimertinib.

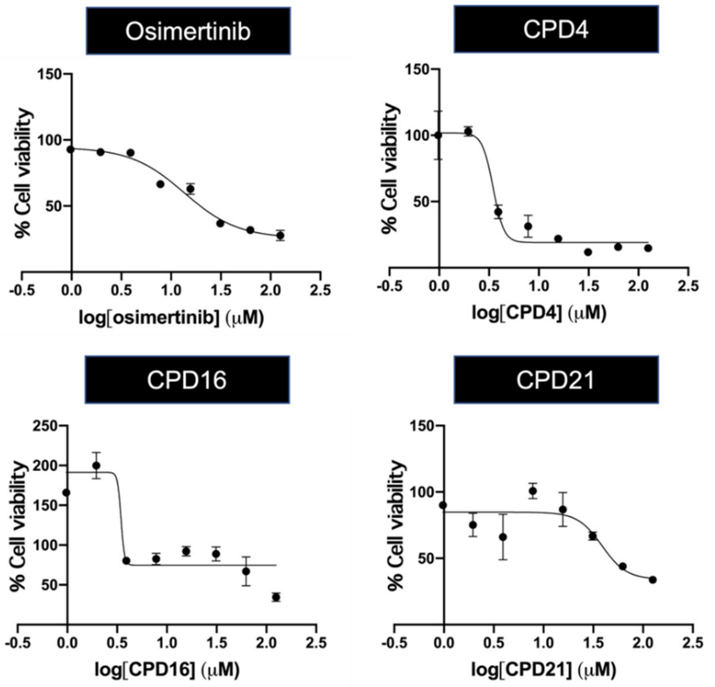

Figure S5: H1975 cell viability against treated screened compounds and osimertinib at different doses. Data were represented as mean±SEM with three replicates.

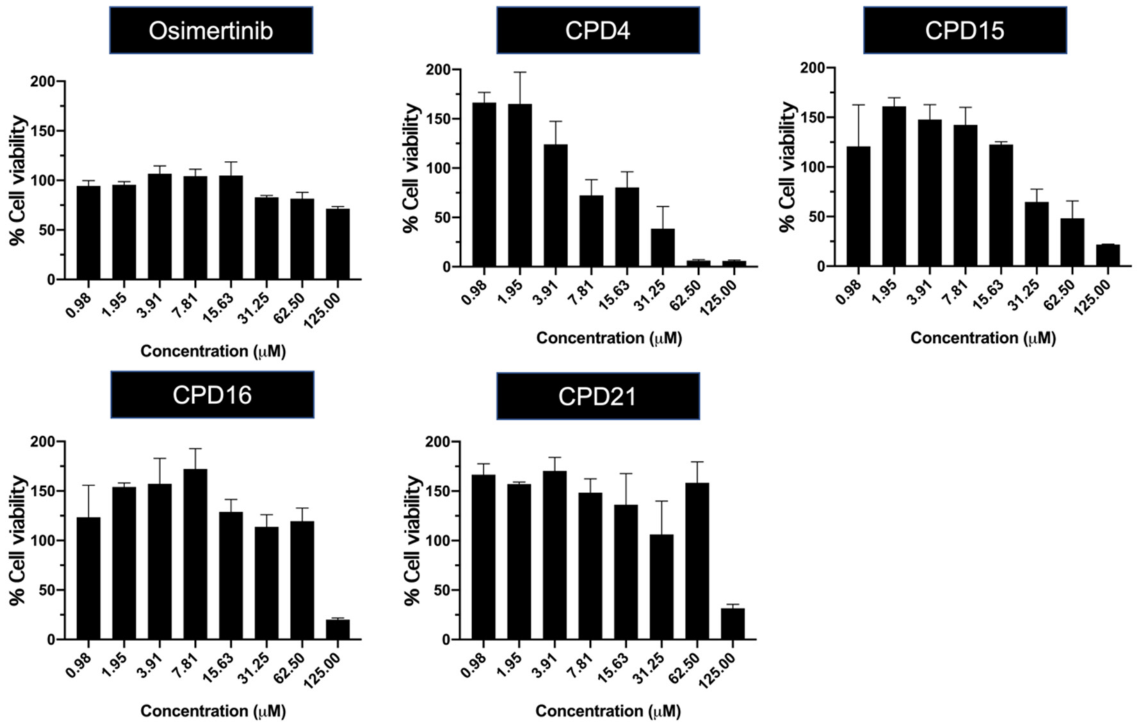

Figure S6: Vero cell viability against treated screened compounds and osimertinib at different doses. Data were represented as mean±SEM with three replicates.
